# The Influence of Preoperative and Postoperative Psychological Symptoms on Clinical Outcome after Shoulder Surgery: A Prospective Longitudinal Cohort Study

**DOI:** 10.1371/journal.pone.0166555

**Published:** 2016-11-15

**Authors:** Rinco C. T. Koorevaar, Esther van ‘t Riet, Marleen J. J. Gerritsen, Kim Madden, Sjoerd K. Bulstra

**Affiliations:** 1 Department of Orthopaedics, Deventer Hospital, Deventer, The Netherlands; 2 Research Department, Deventer Hospital, Deventer, The Netherlands; 3 Department of Psychology, Deventer Hospital, Deventer, The Netherlands; 4 Department of Clinical Epidemiology and Biostatistics, Mc Master University, Hamilton, Canada; 5 Department of Orthopedics, University Medical Center Groningen, University of Groningen, Groningen, The Netherlands; Iranian Institute for Health Sciences Research, ISLAMIC REPUBLIC OF IRAN

## Abstract

**Background:**

Psychological symptoms are highly prevalent in patients with shoulder complaints. Psychological symptoms in patients with shoulder complaints might play a role in the aetiology, perceived disability and pain and clinical outcome of treatment. The aim of this study was to assess whether preoperative symptoms of distress, depression, anxiety and somatisation were associated with a change in function after shoulder surgery and postoperative patient perceived improvement of pain and function. In addition, the change of psychological symptoms after shoulder surgery was analyzed and the influence of postoperative symptoms of psychological disorders after surgery on the change in function after shoulder surgery and perceived postoperative improvement of pain and function.

**Methods and Findings:**

A prospective longitudinal cohort study was performed in a general teaching hospital. 315 consecutive patients planned for elective shoulder surgery were included. Outcome measures included change of Disabilities of the Arm, Shoulder and Hand (DASH) score and anchor questions about improvement in pain and function after surgery. Psychological symptoms were identified before and 12 months after surgery with the validated Four-Dimensional Symptom Questionnaire (4DSQ). Psychological symptoms were encountered in all the various shoulder diagnoses. Preoperative symptoms of psychological disorders persisted after surgery in 56% of patients, 10% of patients with no symptoms of psychological disorders before surgery developed new psychological symptoms. Preoperative symptoms of psychological disorders were not associated with the change of DASH score and perceived improvement of pain and function after shoulder surgery. Patients with symptoms of psychological disorders after surgery were less likely to improve on the DASH score. Postoperative symptoms of distress and depression were associated with worse perceived improvement of pain. Postoperative symptoms of distress, depression and somatisation were associated with worse perceived improvement of function.

**Conclusions:**

Preoperative symptoms of distress, depression, anxiety and somatisation were not associated with worse clinical outcome 12 months after shoulder surgery. Symptoms of psychological disorders before shoulder surgery persisted in 56% of patients after surgery. Postoperative symptoms of psychological disorders 12 months after shoulder surgery were strongly associated with worse clinical outcome.

## Introduction

Shoulder pain and disability is one of the most common musculoskeletal disorders, with a one-year prevalence of 31% in the general population [1]. Shoulder problems are often chronic and may have a substantial impact on physical activity, mental state and quality of life [2,3]. A high prevalence of psychological symptoms in patients with shoulder complaints was reported in several studies [4–6]. Psychological factors in patients with shoulder complaints might play a role in the aetiology, perceived disability and pain, the persistence of symptoms and clinical outcome of treatment. Psychological distress, such as pain anxiety or depression, is increasingly recognised as contributing to pain and disability perception in several musculoskeletal disorders [7,8]. Several studies suggest an important role of somatisation in patients with chronic shoulder pain [9,10].

Identification of psychological symptoms before treatment of shoulder complaints is important because patients who suffer from depression or (pain) anxiety may not be as capable of adapting to and managing painful upper-extremity problems [11]. They may subjectively perceive themselves as more disabled than would be expected on the basis of objective physical findings [12]. Adequate coping mechanisms are important in both the experience of pain and the perception of disability [13]. Biopsychosocial models have recently been proposed, suggesting that a complete understanding of pain-related outcomes will require consideration of psychological and social factors, as well as physical factors [14,15]. In orthopaedic literature, preoperative psychological distress such as depression and anxiety has been reported to be associated with worse clinical outcomes after surgery of the hip, knee and lumbar spine [16,17]. In two recent studies, preoperative psychological distress was not associated with clinical outcome after rotator cuff surgery [18,19]. If, however, preoperative psychological status appears to have an impact on the outcome after shoulder surgery, then the identification and treatment of psychological disorders should be an essential part of the preoperative assessment. After shoulder surgery, the management of stress and pain and the ability to benefit from the rehabilitation program may be influenced by postoperative psychological symptoms. The perception of shoulder pain and disability at follow-up could be affected by psychological symptoms, resulting in inferior clinical outcomes.

The aim of this study was to prospectively assess whether preoperative symptoms of distress, depression, anxiety and somatisation were associated with the change in function after shoulder surgery and postoperative patient perceived improvement of pain and function. The secondary aim was to analyze if psychological symptoms change after shoulder surgery. Finally, we studied the influence of postoperative symptoms of psychological disorders after surgery on the change in function after shoulder surgery and perceived improvement of pain and function. Our hypothesis at the start of this study was that clinical outcome would be less favourable in patients with preoperative psychological symptoms.

## Methods

We used a prospective longitudinal cohort study design for this study.

### Participants

We included all consecutive patients who were scheduled for elective shoulder surgery in a two-and-a-half-year period (January 2011 till June 2013). We followed included patients for 12 months. Surgeries were performed in a general teaching hospital by a single surgeon and his supervised trainees.

We considered patients to be eligible for the study if they were scheduled for elective shoulder surgery and were at least sixteen years of age. Patients were excluded if they were undergoing a diagnostic shoulder arthroscopy or a shoulder arthrodesis, were treated because of a fracture/non-union/mal-union, were unable to complete questionnaires because of language or cognitive disorders or have had a re-operation or sustained a shoulder fracture within the first year of surgery. If patients had more than one operation on one or both shoulders in the study period, only the last operation was included in this study.

### Ethical Approval

The study was carried out in compliance with the principles laid down in the Helsinki Declaration. Participants were informed about the study using a patient information letter and patients had the opportunity to ask questions about the study. Then, informed consent was received orally and formally recorded. When the study was initiated, and approval of the Medical Ethical Committee was obtained, there was no legal requirement for a prospective observational study that used only questionnaires, and no form of intervention for patients, to obtain written informed consent. No children under the age of sixteen were included in the study. Verbal informed consent was obtained from the next of kin, caretakers, or guardians on behalf of the minors enrolled in the study. Approval of this study was obtained from the Regional Medical Ethical Committee Isala Hospital, Zwolle, the Netherlands. Number 14.11151.

### Outcome measures and primary determinant

The primary outcome was the change in functional outcome after shoulder surgery measured with the Disabilities of the Arm, Shoulder and Hand (DASH) score (change of DASH score at baseline and 12-months follow-up). The DASH questionnaire is a 30-item, self-report questionnaire designed to measure physical function and symptoms in people with any musculoskeletal disorder of the upper limb [20]. The DASH has been shown to be reliable, valid and responsive in patients with shoulder disability [21], and has been validated in Dutch for patients with a disorder of the upper limb [22]. The secondary outcome measure was patient perceived improvement of pain and function after shoulder surgery measured 12 months after shoulder surgery with two anchor questions. With an anchor question patients are asked, with a global rating scale, to indicate how much their function (functional anchor) or pain (pain anchor) has changed after surgery [23,24]. The response options of the anchor questions are: completely recovered (7), much improved (6), slightly improved (5), unchanged (4), slightly worse (3), much worse (2) and worse than ever (1).

The primary determinant was the Four-Dimensional Symptom Questionnaire (4DSQ), a psychological questionnaire validated for orthopaedic shoulder patients [4]. The 4DSQ is a 50-item self-report inventory that is able to identify four common psychological symptoms: distress, depression, anxiety and somatisation [25]. The distress scale measures people’s most general and most basic response to stress of any kind, from work or family demands to psychosocial difficulties or life events. The depression and anxiety scales identify specific symptoms of depressive and anxiety disorders, severe enough to warrant treatment. The somatisation scale measures symptoms that are associated with somatic stress. Psychological disorders in this study were defined to be present if patients scored medium or high risk on one of the domains of the 4DSQ [25,26]. The 4DSQ is able to detect depressive and anxiety disorders as well as the Hospital Anxiety Depression Scales (HADS) [26] and an English version of the 4DSQ has been validated [27].

### Data collection

Prior to elective shoulder surgery, orthopaedic patients were seen at an outpatient clinic by an independent physiotherapist two to three weeks before surgery. The following preoperative demographic and clinical variables were collected: age, gender, dominant shoulder, duration of shoulder complaints, and whether the patient has a history of surgery on the same shoulder. The DASH score, VAS pain in rest and during activity and 4DSQ test were obtained.

After surgery the following data were recorded: type of operation and whether the right or left shoulder was operated on. After one year data were obtained using a web-based system. The patients completed an online questionnaire at home containing the DASH score, VAS pain at rest and during activity, 4DSQ score (for patients who underwent surgery in 2012 and 2013) and the two anchor questions about change in pain and function. The 4DSQ score was not obtained 12 months after surgery in patients who underwent surgery in 2011. We started this study in 2011 and at the end of 2011 we changed our study protocol and started to collect psychological symptoms with the 4DSQ also 12 months after surgery. If the patient didn’t respond to our request to complete the postoperative questionnaire, a telephone interview by an independent research nurse was conducted that included the two anchor questions about pain and function. All data were collected independently from the clinical team by the research unit of our Orthopedic Department, using standardized case report forms and a study-specific database. The 4DSQ scores were unknown to the clinical physicians to minimize bias.

### Statistical analysis

Descriptive analyses, including frequency counts and percentages, were calculated for all data gathered. Data are presented as percentage or mean (SD). Differences between baseline and follow-up were tested using Wilcoxon tests for continuous variables and chi-square tests for categorical variables. No statistical imputation process was undertaken for missing information (i.e. we used complete case analysis).

The change in DASH score (continuous) was used as the dependent variable. For all four domains of the 4DSQ (distress, depression, anxiety and somatisation) we performed multivariable logistic regression analyses with the preoperative and the postoperative psychological domain (dichotomous yes/no) as the primary determinant and the change of DASH score as the dependent variable. To investigate whether there is an independent relationship between psychological disorders and functional outcome, the following clinically relevant potential confounders were added to the regression models: age, gender, dominant shoulder, duration of symptoms, history of previous shoulder surgery and preoperative DASH score. These confounders were added to the regression model one by one and the effect of the variable on the relation between psychological domain and change of DASH score was studied. If the regression coefficient of this relation was altered 10% or more after adding the variable, the variable was included into the final model as confounding factor. In addition, models with preoperative psychological disorders as the primary determinant were adjusted for postoperative psychological disorders and the other way around. Risk of multicollineairity was checked using variance inflation factor statistics. A variance inflation factor of over 5 was considered a risk for multicollinearity. Effect modification of gender was checked by including an interaction term into the model. SPSS statistical software (version 22.0; IBM, Armonk, NY, USA) was used for data management and statistical analyses.

## Results

### Description of the study population

**Fig 1:**
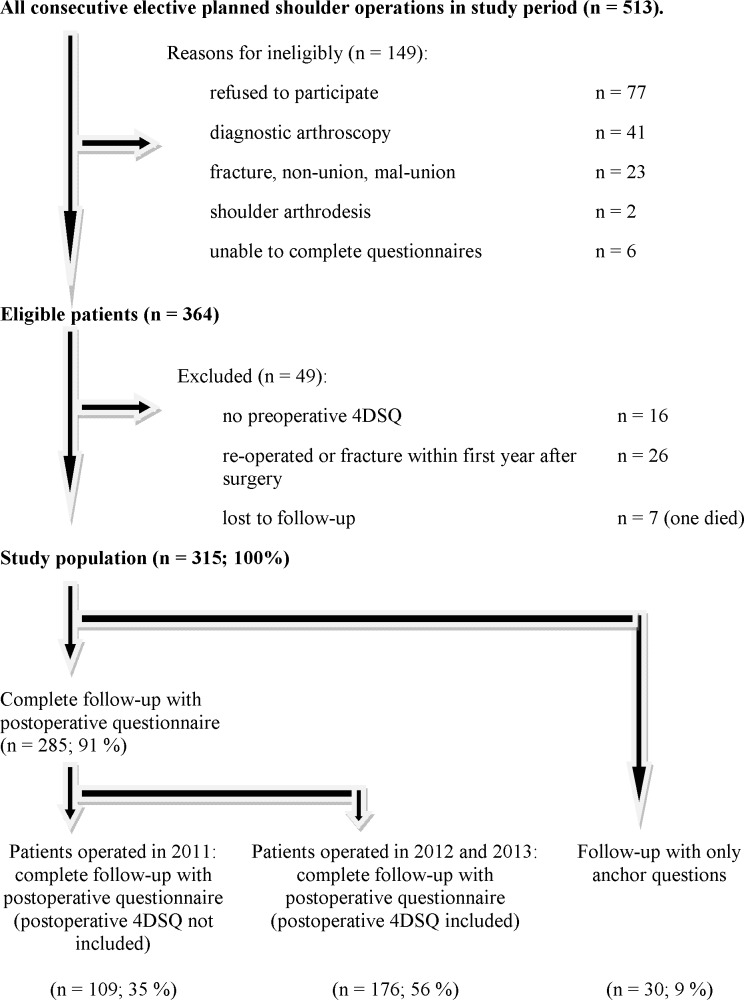
Flow diagram with study enrolment and follow-up.

Fig 1 shows a flow diagram with study enrolment and follow-up. 315 patients were included in this study. Of the 315 patients, 285 patients completed the postoperative questionnaire and 30 patients were interviewed by phone, including the anchor questions. The answers to the anchor questions about improvement of pain and function were not significantly different between these two groups. The postoperative questionnaire of patients who had surgery in 2011 did not include the 4DSQ score. In 2012 and 2013 the postoperative 4DSQ score was scored 12 months after surgery and (completed by 176 patients). Baseline characteristics and outcome measures were not statistically significantly different between the group with postoperative 4DSQ scores and the group with no postoperative 4DSQ scores 12 months after surgery. Demographic and clinical data at baseline and 12 months after surgery are presented in Table 1. A significant improvement in DASH score and VAS pain (during activity) was observed after surgery. Before shoulder surgery, distress was identified in 20% of the patients, depression in 7%, anxiety in 13% and somatisation in 15%. Symptoms of psychological disorders were encountered in all the various shoulder diagnoses (Table 2). In 56% of patients with symptoms of preoperative psychological disorders, symptoms of psychological disorders persisted 12 months after surgery (distress in 57%, depression in 75%, anxiety in 41%, somatisation in 65%).

**Table 1:** Demographic and clinical data of the study population (n = 315).

	Baseline	Follow-up 12 months	P value
**Mean age and std. dev. (yr)**	52 (16)		
**Male gender (no. [%])**	167 (53)		
**Dominant shoulder (no. [%])**	198 (63)		
**Duration of symptoms (months; SD)**	32 (40)		
**History of surgery (no. [%])**	32 (10)		
**Distress**			
**symptoms (mean (SD))**	5.9 (5.8)	5.2 (7.0)*	0,06
**medium/high risk (no. [%])**	20 (20%)	18%*	
**Depression**			
**symptoms (mean (SD))**	0.5 (1.6)	0.89 (2.1)*	0,01
**medium/high risk (no. [%])**	24 (7%)	15%*	
**Anxiety**			
**symptoms ((mean (SD))**	1.1 (2.0)	1.2 (2.9)*	0,44
**medium/high risk (no. [%])**	43 (13%)	13%*	
**Somatisation**			
**symptoms ((mean (SD))**	6.4 (5.0)	6.4 (6.2)*	0,82
**medium/high risk (no. [%])**	46 (15%)	18%*	
**Mean (SD) VAS pain in rest**	1.7 (2.2)	1.6 (2.2)	0,08
**Mean (SD) VAS pain during activity**	7.9 (5.1)	2.5 (2.6)	<0.001
**Mean DASH score and std. dev.**	40.5 (20.9)	16.8 (17.0)	<0.001
**Anchor question pain**			
**mean and std. dev.**		6.1 (0.9)	
**≥ 5**		92%	
**Anchor question function**			
**mean and std. dev.**		6.0 (1.1)	
**≥ 5**		91%	

* n = 176

**Table 2:** Shoulder diagnosis and preoperative symptoms of psychological disorders.

Diagnosis		Distress	Depression	Anxiety	Somatisation
**Subacromial pain syndrome (n = 39)**	(no. [%])	8 (21%)	4 (10%)	6 (15%)	9 (23%)
**Rotator cuff rupture (n = 88)**	(no. [%])	18 (20%)	7 (8%)	6 (7%)	10 (11%)
**Instability (n = 67)**	(no. [%])	8 (12%)	2 (3%)	6 (9%)	7 (10%)
**Acromioclavicular osteoarthritis (n = 34)**	(no. [%])	7 (21%)	2 (6%)	5 (15%)	6 (18%)
**Glenohumeral osteoarthritis (n = 43)**	(no. [%])	10 (23%)	4 (9%)	11 (26%)	7 (16%)
**Other shoulder surgery (n = 44)**	(no. [%])	15 (34%)	5 (11%)	9 (20%)	7 (16%)
**Total (n = 315)**	(no. [%])	66 (20%)	24 (7%)	43 (13%)	46 (15%)
**P value**		0.19	0.56	0.10	0.72

P value presents the comparison of the prevalence of each symptom of psychological disorder separately across the different types of diagnosis.

### Primary outcome measure: change of DASH score

A significant improvement in DASH score one year after surgery was observed in patients with and without symptoms of psychological disorders before surgery (Table 3). Preoperative symptoms of psychological disorders had a negative impact on the self-assessed DASH scores, resulting in higher DASH scores before surgery. Multivariable logistic regression analyses were performed with preoperative symptoms of psychological disorders as the independent variable and change of DASH score as the dependent variable (Table 4). Distress, depression, anxiety and somatisation before surgery, after adjustment for age, gender and DASH score preoperatively, predicted less of an improvement in DASH score. When additionally adjusted for postoperative symptoms of psychological disorders, the regression coefficients dropped considerably and all significant associations disappeared (Table 4).

**Table 3:** DASH scores in patients with and without preoperative symptoms of psychological disorders.

Preoperative symptoms of psychological disorders		DASH preop	DASH postop	DASH change score
**distress**	n = 66 (20%)	51.6	26.2	26.5
**no distress**	n = 249 (80%)	37.7	14.5	23.0
**depression**	n = 24 (7%)	50,9	31.2	16.7
**no depression**	n = 291 (93%)	39.7	15.7	24.2
**anxiety**	n = 43 (13%)	51.0	28.5	21.9
**no anxiety**	n = 272 (87%)	38.9	15.1	23.9
**somatisation**	n = 46 (15%)	52.1	30.2	21.8
**no somatisation**	n = 269 (85%)	38.6	14.6	24.0

**Table 4:** Association of preoperative symptoms of psychological disorders with clinical outcome.

	Change of DASH score	Anchor question pain	Anchor question function
Preoperative Distress			
Model 1			
Coefficient	-8.06	-0.39	-0.36
95% CI	-12.96 - -3.16	-0.67 - -0.10	-0.67 - -0.04
P value	0.001	0.008	0.03
Preoperative Distress			
Model 2			
Coefficient	0.30	0.31	0.19
95% CI	-6.09–6.7	-0.12–0.74	-0.31–0.69
P value	0.93	0.16	0.45
Preoperative Depression			
Model 1			
Coefficient	-15.15	-0.74	-0.62
95% CI	-22.54 - -7.76	-1.17 - -0.31	-1.09 - -0.14
P value	< 0.001	0.001	0.011
Preoperative Depression			
Model 2			
Coefficient	-4.68	0.09	0.07
95% CI	-14.72 - -5.36	-0.56–0.74	-0.68–0.82
P value	0.36	0.78	0.85
Preoperative Anxiety			
Model 1			
Coefficient	-9.51	-0.55	-0.56
95% CI	-15.21 - -3.81	-0.88 - -0.23	-0.92 - -0.19
P value	0.001	0.001	0.003
Preoperative Anxiety			
Model 2			
Coefficient	-6.25	-0.27	-0.33
95% CI	-13.84–1.30	-0.75–0.21	-0.89–0.23
P value	0.10	0.27	0.24
Preoperative Somatisation			
Model 1			
Coefficient	-11.84	-0.59	-0.57
95% CI	-17.29 - -6.40	-0.90 - -0.26	-0.93 - -0.21
P value	< 0.001	0.001	0.002
Preoperative Somatisation			
Model 2			
Coefficient	-3.00	-0.12	-0.12
95% CI	-10.53–4.52	-0.62–0.37	-0.68–0.45
P value	0.43	0.63	0.69

Model 1: adjusted for age, gender and preoperative DASH score in the models with DASH change score as outcome. In the models with the anchor questions, the models were additionally adjusted for previous surgery.

Model 2: Model 1 + also adjusted for postoperative symptoms of psychological disorders

### Secondary outcome measures: anchor question pain and function

Multivariable regression analyses (Table 4), with adjustment for age, gender, history of shoulder surgery and DASH score preoperatively, showed that all preoperative symptoms of psychological disorders were negatively associated with improvement of pain and function after shoulder surgery. Inclusion of postoperative symptoms of psychological disorders as possible confounders explained the significant association between preoperative symptoms of psychological disorders and the improvement in pain and function. When additionally adjusted for postoperative symptoms of psychological disorders, all significant associations disappeared.

### Influence of postoperative symptoms of psychological disorders on clinical outcome after shoulder surgery

**Fig 2:**
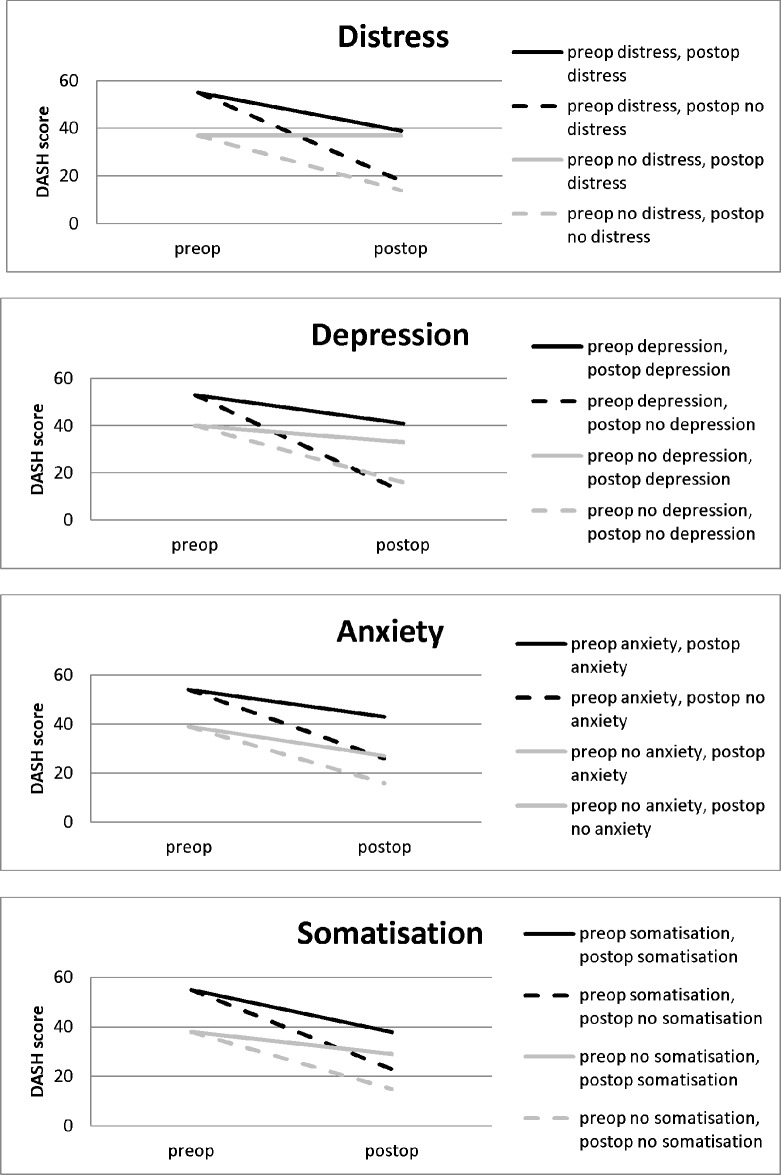
Influence of symptoms of psychological disorders on the DASH scores before and after surgery.

Data for 176 patients with pre- and postoperative 4DSQ scores were analysed. Postoperative symptoms of psychological disorders were highly significant predictors of worse clinical outcome (Table 5). The change of DASH score was significantly decreased when postoperative symptoms of psychological disorders were present and perceived improvement of pain and function after shoulder surgery was less favourable, compared to patients with no postoperative symptoms of psychological disorders. With the adjustment of preoperative symptoms of psychological disorders as possible confounders in the model, postoperative symptoms of psychological disorders remained an independent predictor of worse clinical outcome after shoulder surgery. These negative associations were observed for all symptoms of psychological disorders in relation to the change of DASH score. Postoperative distress and depression were associated with worse perceived improvement of pain and postoperative distress, depression and somatisation were associated with worse perceived improvement of function. Fig 2 illustrates the influence of preoperative and postoperative symptoms of psychological disorders on the DASH scores before and after surgery.

**Table 5:** Association of postoperative symptoms of psychological disorders with clinical outcome.

	Change of DASH score	Anchor question pain	Anchor question function
Postoperative Distress			
Model 1			
Coefficient	-20.49	-0.81	-0.89
95% CI	-26.41 - -14.57	-1.20- -0.41	-1.35- -0.43
P value	<0.001	<0.001	<0.001
Postoperative Distress			
Model 2			
Coefficient	-20.63	-0.95	-0.98
95% CI	-27.25 - -14.00	-1.39- -0.51	-1.49- -0.47
P value	<0.001	<0.001	<0.001
Postoperative Depression			
Model 1			
Coefficient	-18.07	-0.76	-0.91
95% CI	-24.61 - -11.53	-1.19- -0.34	-1.40- -0.42
P value	<0.001	0.001	<0.001
Postoperative Depression			
Model 2			
Coefficient	-16.59	-0.79	-0.93
95% CI	-23.86 - -9.32	-1.26- -0.32	-1.47- -0.38
P value	<0.001	0.001	0.001
Postoperative Anxiety			
Model 1			
Coefficient	-13.48	-0.54	-0.58
95% CI	-20.70 - -6.26	-1.00- -0.08	-1.1- -0.04
P value	<0.001	0.02	0.04
Postoperative Anxiety			
Model 2			
Coefficient	-11.62	-0.46	-0.47
95% CI	-19.15 - -4.10	-0.94–0.19	-1.03- -0.08
P value	0.003	0.06	0.10
Postoperative Somatisation			
Model 1			
Coefficient	-15.68	-0.42	-0.67
95% CI	-21.67 - -9.69	-0.82- -0.03	-1.12- -0.22
P value	<0.001	0.04	0.004
Postoperative Somatisation			
Model 2			
Coefficient	-14.37	-0.37	-0.62
95% CI	-21.23 - -7.51	-0.82–0.83	-1.13- -0.10
P value	<0.001	0.11	0.02

Model 1: adjusted for age, gender and preoperative DASH score in the models with DASH change score as outcome. In the models with the anchor questions, the models were additionally adjusted for previous surgery.

Model 2: Model 1 + also adjusted for preoperative psychological disorders

## Discussion

The findings of this study suggest that preoperative symptoms of distress, depression, anxiety and somatisation were not associated with a worse clinical outcome after shoulder surgery. Symptoms of psychological disorders before shoulder surgery persisted in the majority of patients. Postoperative symptoms of postoperative psychological disorders, however, were a strong predictor of worse clinical outcome.

In the study period most patients reported pain relief and better function after shoulder surgery but psychological symptoms did not change after shoulder surgery in most patients. Operative treatment of shoulder pathology seems not to be associated with improvement of psychological symptoms. Prevention of psychological symptoms was not the aim of this study. Future studies could focus on the prevention of psychological symptoms after shoulder surgery, for example by cognitive treatment, and observe the effect on clinical outcome. Two recent studies, with small study populations, evaluated preoperative psychological distress and functional outcome after rotator cuff surgery [18,19]. Their results are in line with the results found in this study that preoperative symptoms of psychological disorders were not negatively associated with functional outcome after shoulder surgery. This is the first paper, to our knowledge, that observed that postoperative symptoms of psychological disorders were a strong predictor of worse clinical outcome. The prevalence of symptoms of psychological distress was lower at follow-up in these two studies, in contrast to our study population. We observed a disappearance of symptoms of psychological disorders in only 42% of the patients 12 months after surgery, but 10% of patients with no symptoms of psychological disorders before surgery developed new psychological symptoms. In the study by Potter et al. preoperative psychological distress disappeared in 54% of patients after surgery, 5% of patients with no preoperative psychological distress became distressed after shoulder surgery [18]. Recently, biopsychosocial models have been proposed, suggesting that a complete understanding of pain-related outcomes will require consideration of psychological and social factors, as well as physical and genetic factors [14]. George et al. investigated whether genetic and psychological factors could predict 12-months post-operative pain and disability after shoulder surgery [15]. Strong statistical evidence was observed between post-operative outcome and the combination of genes involved with inflammation (IL6) with pain catastrophizing and genes involved with inflammation (IL6) with depressive symptoms [15]. They recommended that the interaction between genetic and psychological factors should be further explored in future studies to better understand the presence of persistent post-operative pain after shoulder surgery and investigate the effectiveness of individual treatment.

We observed that preoperative DASH scores of patients with preoperative symptoms of psychological disorders were significantly higher than in patients with no symptoms of psychological disorders. It seems that the perception of functional disability, activity level, and pain is negatively influenced by symptoms of psychological disorders, which leads to higher DASH scores. This phenomenon has been observed in other studies and also found in other PROMs, such as the Simple Shoulder Test [6,8,11]. In this study we used anchor questions as specific questions about pain and functional improvement after surgery. Patients are asked in a single question to indicate how much their function or pain has changed since baseline. The anchor questions may be affected by recall bias [28]. Tashjian classified these anchor questions as retrospective assessment of improvement of outcome [29]. He compared prospective and retrospective assessment of functional outcome after rotator cuff repair. Retrospective assessment had fair correlations with prospective determined improvement, for example, with the DASH score. Patient satisfaction was more highly correlated with retrospective evaluations than with the prospective improvement in functional outcome measures.

### Strengths and limitations

The major strength of our study is the pragmatic prospective longitudinal design. This study was conducted in an everyday clinical setting with a large sample size. We used a validated orthopaedic clinical outcome questionnaire and a psychological questionnaire validated for orthopaedic shoulder patients.

Some limitations have to be mentioned. The DASH score can be influenced by any disorder of the upper extremity; not just the shoulder disorder of interest in this study. Subjective factors such as pain or depression have been reported to have a greater influence when disability is measured with respect to the entire arm [30]. There may be other factors, like comorbidities, workers’ compensation status, work status, education level, marital status and smoking that may affect DASH scores. We did not include these factors in our study protocol. Postoperative 4DSQ scores were not included in the online postoperative questionnaire in 2011 (139 patients). After changing the study protocol at the end of 2011 we started to collect postoperative psychological symptoms in all patients who underwent surgery in 2012 and 2013 by including the 4DSQ in the online postoperative questionnaire (176 patients). Psychological disorders in this study were defined to be present if the patients scored medium or high risk on one of the domains of the 4DSQ. The 4DSQ questionnaire is a tool to identify psychological symptoms. However, having significant psychological symptoms is not the same as having a psychological illness. Psychological illnesses have to be diagnosed by a psychologist or psychiatrist using DSM-V criteria. We didn’t account for the use of antidepressants and whether patients were being treated by a psychologist or psychiatrist. Psychological symptoms may be associated with medical comorbidities. We did not include medical comorbidities in our study protocol. We included a heterogeneous study population with shoulder complaints that had different diagnoses, surgery procedures, pain levels, functional disability and duration of symptoms. The aim of this study was to analyze the association between psychological disorders and clinical outcome in all patients with shoulder pain and disability that were treated with shoulder surgery. Psychological disorders were observed in all various shoulder diagnoses before surgery. Future studies should investigate if the results of this study are confirmed in all various shoulder surgical procedures. Complications of the surgical procedures were not registered during the study period. We did not calculate the number of patients needed before the start of our study. At the start of the study no data from adequate studies were available on which to base our calculations.

## Conclusion and Clinical Relevance

Preoperative symptoms of distress, depression, anxiety and somatisation were not associated with a worse clinical outcome 12 months after shoulder surgery. Symptoms of psychological disorders before shoulder surgery persisted in the majority of patients. Postoperative symptoms of psychological disorders 12 months after shoulder surgery, however, were a strong predictor of worse clinical outcome.

As a consequence of the findings in this study, we suggest that patients with symptoms of psychological disorders before surgery should not be excluded from shoulder surgery. Most patients with psychological symptoms before surgery benefit from shoulder surgery, especially when psychological symptoms disappear after surgery. Postoperative symptoms of psychological disorders were strong predictors of a worse clinical outcome. In order to interpret clinical outcome after shoulder surgery, the influence of psychological symptoms on the clinical outcome should be taken into account.
